# Simultaneous Extraction and Analysis of Seven Major Saikosaponins from *Bupleuri* Radix and the Exploration of Antioxidant Activity and Its Mechanism

**DOI:** 10.3390/molecules28155872

**Published:** 2023-08-04

**Authors:** Ning Wang, Qian Li

**Affiliations:** State Key Laboratory of Aridland Crop Science, College of Agronomy, Gansu Agricultural University, Lanzhou 730070, China; wning_1207@163.com

**Keywords:** *Bupleuri* Radix, saikosaponins, ultrasound-assisted extraction, antioxidant, network pharmacology

## Abstract

Saikosaponins (SS) are the main active components of *Bupleuri* Radix. In this study, the yields of SS a, b_1_, b_2_, c, d, e, and f were simultaneously determined using the HPLC-DAD dual wavelength method, and the ultrasound-assisted extraction process of saikosaponins was optimized using the response surface methodology. The antioxidant effect of saikosaponins was investigated using the scavenging rate of 1, 1-diphenyl-2-picrylhydrazyl (DPPH), 2, 2-diazo-bis (3-ethyl-benzothiazole-6-sulfonic acid) diammonium salt (ABTS), and hydroxyl (-OH) groups, and the mechanism was clarified via network pharmacological analysis. The results showed that the optimal extraction process of SS was a 5% ammonia–methanol solution as an extraction solvent, a material–liquid ratio of 1:40, a temperature of 46.66 °C, an extraction time of 65.07 min, and an ultrasonic power of 345.56 W. The total content of the seven saikosaponins under this condition was up to 6.32%, which was close to the model’s predicted value of 6.56%, where the yields of the seven saikosaponins a, b_1_, b_2_, c, d, e, and f were 1.18%, 0.11%, 0.26%, 1.02%, 3.02%, 0.38%, and 0.44%, respectively. The saikosaponins have an obvious scavenging ability for DPPH, ABTS, and -OH radicals. The interactions of seven saikosaponins with antioxidant targets were studied, and a database was used to collate the core of saikosaponins and antioxidants through network pharmacology. The mechanisms of the antioxidant effects of the saikosaponins were derived via GO enrichment analysis and KEGG pathway analysis. Finally, the binding energy of the saikosaponins to the antioxidant targets was found to be less than −5.0 kcal·mol^−1^ via molecular docking, indicating that the antioxidant capacity of the saikosaponins are good. Therefore, this study developed a rapid and efficient method for the extraction of saikosaponins, which provides a theoretical basis for an in-depth understanding of the rational utilization of saikosaponins and the development of their medicinal value.

## 1. Introduction

*Bupleuri* Radix has a 2000-year history of medicinal use, and its first published use was in the Shennong Ben Cao Jing [1]. As a commonly used clinical herb, *Bupleuri* Radix can de-stress and protect the liver, promote yang energy, expel evil, and relieve fever [2]. Saikosaponins (SS) are a class of saponin derivatives with biological activity [1], mainly including SSa, SSb_1_, SSd, SSc, SSs_1_, SSb_2_, SSv, SSv_1_, SSv_2_, SSt, SSb_3_, SSe, SSf, SSq_1_, SSq_2_, etc. SSd is the most abundant and biologically active saikosaponin, and SSa is the second most productive and biologically active [3,4,5,6]. Saikosaponins have various pharmacological effects such as antipyretic, analgesic, cough suppressant, sedative, anti-inflammatory, antiepileptic, antiviral, antitumor, and hepatoprotective properties [1,7,8]. It is evident that saikosaponins have significant biological activities and show great potential in medicine.

Therefore, establishing an efficient extraction and isolation method for saikosaponins is of great importance.

Ultrasonic- assisted extraction uses ultrasonic cavitation and thermal effects to destroy the cell walls of plants, thus enhancing the rapid entry of a solvent into the cells for extraction [9], which has the advantages of short extraction times, high yields, and no heating, etc. The ultrasonic-assisted solvent extraction of plant secondary metabolites has been widely used in the food, chemical, and medical fields [10]. There are more studies on the ultrasound-assisted extraction of total saikosaponins, but fewer studies have been reported on the simultaneous determination of saikosaponins a, b_1_, b_2_, c, d, e, and f. Response surface methodology (RSM) is frequently applied as an advanced chemometric tool for the optimization of extraction processes [11]. RSM designs can reduce the number of experiments and produce a mathematical model to account for the interaction of various independent variables, and a Box–Behnken design (BBD) is one type of RSM which is easier to interpret and implement compared to other methods [12]. Therefore, the ultrasound-assisted extraction method combined with response surface methodology to optimize the extraction process of saikosaponins is essential to improving the utilization of *Bupleuri* Radix.

In general, the body can maintain a mutually balanced relationship between oxidation and antioxidation, but sometimes, oxidative stress can occur and lead to pathologies in the body, such as atherosclerosis, osteoarthritis, cancer, and other diseases [13,14,15,16]. Studies have shown that saikosaponins have some scavenging ability for both DPPH and ABTS free radicals [17] and can participate in some pathways to reduce oxidative stress in the body [1,18], thus acting as an antioxidant. Network pharmacology was proposed in 2007 by Hopkins, a British pharmacologist, to explore the mechanism of drug action in diseases through studying the interactions of drugs and their chemical components with their targets [19]. Network pharmacology is an important part of systems biology and bioinformatics which focuses on the intervention and regulation of diseases by multiple components in a single herbal medicine or compound [20] and has the role of discovering multi-level, multi-component, multi-target, and interacting herbal networks [21] and predicting the active ingredients or components under potential biological mechanisms [22]. Yang et al. [23] used DPPH free radical scavenging to assess the antioxidant activity of *Schisandra chinensis* by using network pharmacology to validate its antioxidant mechanism of action. Wang et al. [24] determined the antioxidant activity of rhubarb seeds via DPPH, ABTS, and FRAP antioxidant assays and used network pharmacology combined with molecular docking techniques to further screen their antioxidant-related targets. Therefore, the determination of saikosaponins’ antioxidant mechanism of action through network pharmacology based on their free radical scavenging effects would provide a theoretical basis for the development of saikosaponins as antioxidants for modern medicine and the health care industry.

In this study, the ultrasound-assisted extraction of seven major saikosaponins was developed. The content of saikosaponins a, b_1_, b_2_, c, d, e, and f were simultaneously determined using the HPLC-DAD dual wavelength method. Furthermore, the scavenging rates of saikosaponins on DPPH, ABTS, and -OH were studied to determine their antioxidant activity, and their antioxidant mechanism of action was validated using network pharmacology, which provides a theoretical basis for the development of saikosaponins as antioxidants for modern medicine and the health care industry. This work aims to establish an efficient and rapid extraction method for saikosaponins, which could provide theoretical guidance for the industrial extraction and functionalization of saikosaponins. This work is innovative in its simultaneous determination of the seven saikosaponins using the HPLC-DAD dual wavelength method and study of their antioxidant mechanism.

## 2. Results and Discussion

### 2.1. The Determination of Seven Saikosaponins via HPLC-DAD

**Figure 1:**
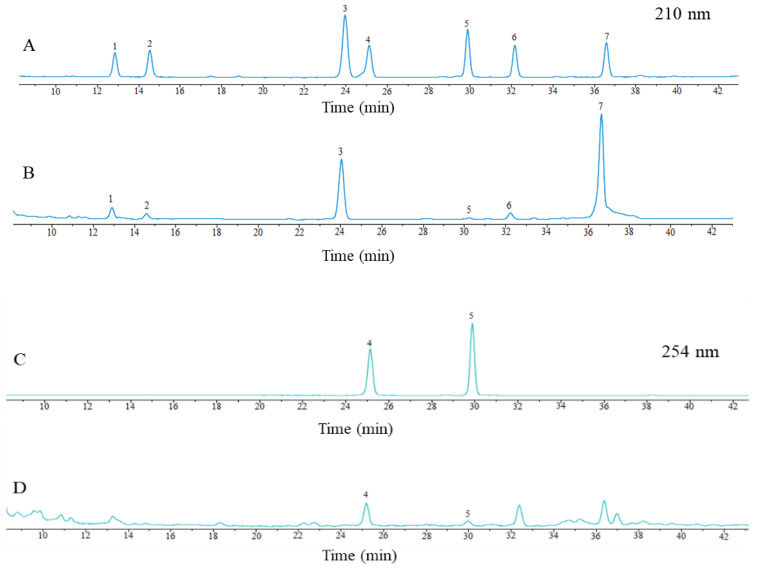
HPLC chromatographic graphs of mixed controls (**A**,**B**) and samples (**C**,**D**). Notes: 1 is SSc; 2 is SSf; 3 is SSa; 4 is SSb_2_; 5 is SSb_1_; 6 is SSe; 7 is SSd.

The results of HPLC chromatographic graphs for the analysis of the seven saikosaponins are shown in Figure 1. SSa, SSc, SSd, SSe, and SSf were separated well at a wavelength of 210 nm, and SSb_1_ and SSb_2_ were separated well at 254 nm. The contents of saikosaponins a, b_1_, b_2_, c, d, e, and f were 1.18%, 0.11%, 0.26%, 1.02%, 3.02%, 0.38%, and 0.44%, respectively, which indicates that the chromatographic conditions can be good for the separation of the seven saikosaponins.

### 2.2. Results of Linearity Relationship and Methodological Validation

As can be seen from Table 1, the standard curve equations of the seven saikosaponins were within the linear range of 7.413~71.413 μg·mL^−1^, with an R^2^ greater than 0.999, indicating that the stability of the method and apparatus for the isolation of saikosaponins was good. As shown in Table 2, the RSD values of the precision, stability, and reproducibility of the apparatus and method are less than 5%, indicating good precision and reproducibility. The samples could be stable within 24 h.

### 2.3. Effect of Different Solvents on the Yields of Saikosaponins

**Figure 2:**
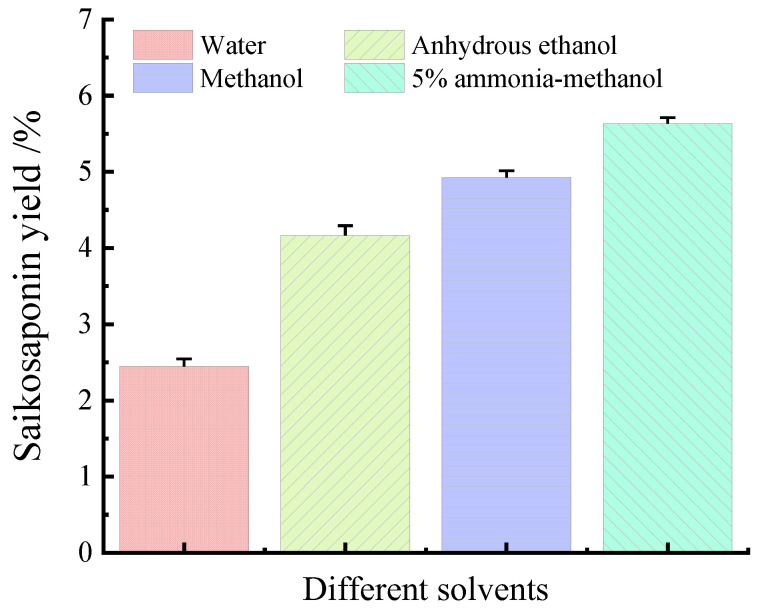
Effect of different solvents on the yields of saikosaponins.

The effects of water, anhydrous ethanol, methanol, and 5% ammonia–methanol solution on the yields of saikosaponins were compared under the same conditions. As shown in Figure 2, the extraction yields of the four solvents were 2.47%, 4.03%, 4.84%, and 5.60%, respectively, which shows that the 5% ammonia methanol solution has the highest extraction yields of saikosaponins, which is in agreement with the results of Sun et al. [25]. Therefore, the 5% ammonia–methanol solution can be used as the best solvent for the extraction of saikosaponins.

### 2.4. Effect of Different Extraction Temperatures on the Yields of Saikosaponins

**Figure 3:**
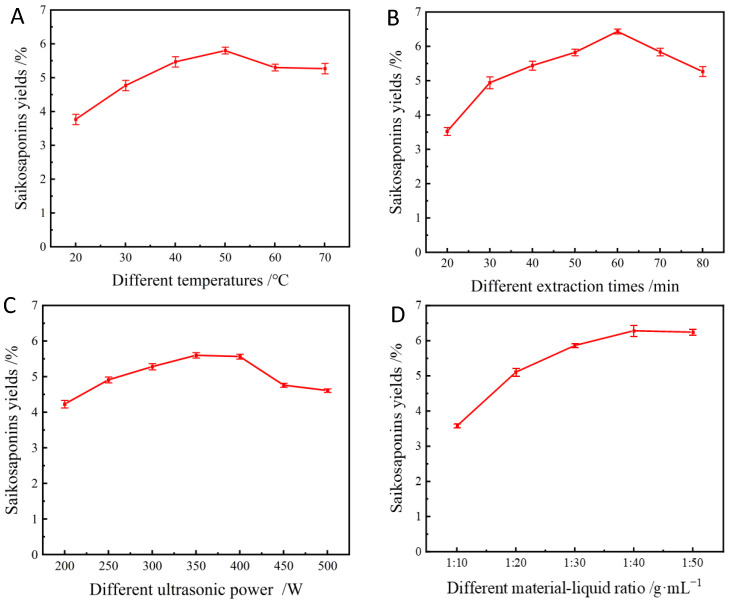
Effect of extraction temperature (**A**), extraction time (**B**), sonication power (**C**), and material–liquid ratio (**D**) on the yields of saikosaponins.

Several sets of experiments were conducted to comprehensively study the yields of saikosaponins at different temperatures. The extraction was carried out at 20 °C, 30 °C, 40 °C, 50 °C, 60 °C, and 70 °C, and the extraction yields were counted and analyzed, which are shown in Figure 3A. The yields of saikosaponins increased and then decreased slightly to 3.76%, 4.73%, 5.46%, 5.80%, 5.30%, and 5.27% with the increase in temperature. It can be seen that the highest extraction yields of saikosaponins were obtained when the temperature was 50 °C. This may be due to the fact that the diffusion of the compounds is faster at the appropriate temperature (50 °C), which increases the extraction rate. However, the high temperature causes the decomposition or denaturation of saikosaponins, which leads to a decrease in the extraction rate [26]. Therefore, in order to ensure extraction yields and control energy consumption, the extraction temperature of 50 °C was chosen as the reference temperature for the subsequent experiments.

### 2.5. Effect of Different Extraction Times on the Yields of Saikosaponins

As shown in Figure 3B, increasing the extraction time from 20 min to 60 min resulted in a gradual increase in the saikosaponins’ yields, with the highest extraction yield being 6.31% at 60 min. However, a further increase in extraction time significantly reduced the extraction yields of saikosaponins. As reported by Tomšik et al. [27], prolonging the extraction time extends the contact time between the compounds and the solvent, which facilitates the diffusion of the target compounds. However, prolonged extraction is not applicable to bioactive compounds, which may lead to the degradation of the products [28]. In this study, an extraction time of 60 min was chosen as the optimum extraction time for the extraction of saikosaponins.

### 2.6. Effect of Different Ultrasonic Power on the Yields of Saikosaponins

The ultrasonic power was varied between 200 and 500 W, as shown in Figure 3C. The results showed that when the ultrasonic power increased from 200 W to 400 W, the extraction yields of saikosaponins increased from 4.24% to 5.60%, and then the saikosaponins yields decreased to 4.56% as the ultrasonic power gradually increased to 500 W. This is because when the ultrasonic power is too high, it accelerates the drift during the extraction process, which leads to the accelerated generation of bubbles in the solvent, resulting in the wall-breaking effect and the decreased dissolution rate of the saikosaponins [29]. Thus, considering the high yields of saikosaponins and relatively low energy consumption, 350 W was considered as the optimal ultrasonic power.

### 2.7. Effect of Different Material–Liquid Ratios on the Yields of Saikosaponins

As can be seen from Figure 3D, the extraction yields of saikosaponins increased as the material–liquid ratio increased from 1:10 to 1:30, and the maximum yields of the saikosaponins reached 6.28% when the material–liquid ratio was 1:40; however, the extraction yields decreased as the liquid–solid ratio further increased, which was consistent with the results of a previous study [30]. It has been shown that the larger the concentration gradient of the solid–liquid ratio, the faster the mass transfer from solids to solvent. Nevertheless, this effect is diminished if the liquid–solid ratio is too high because the main mass transfer is limited to the liquid–solid ratio range to avoid solvent wastage and extraction costs [29]. Therefore, the optimum liquid–solid ratio of 1:40 was selected for further study.

### 2.8. Response Surface Design and Experimental Analysis

#### 2.8.1. Model Fitting and Data Analysis

Response surface optimization analysis was performed using Design-Expert.V8.0.6.1 software for multiple linear regression and binomial equation fitting of the factors (extraction temperature, time, and power) affecting the yields of saikosaponins. The experimental results are shown in Table 3, and the fitted equations are as follows.

Saikosaponins yields/% = 6.50 − 0.17A + 0.11B − 0.071C + 0.14AB − 0.088AC + 0.041BC −0.17A^2^ − 0.043B^2^ − 0.11C^2^

The regression model was further evaluated with ANOVA and significance tests, and the results are presented in Table 4. The F-value and *p*-value can be used for significance assessment, where the higher the F-value and the lower the *p*-value, the higher the significance [31]. From Table 4, the linear regression equation has an F-value of 15.81, a sum of squares of 0.68, a degree of freedom of 9, and a mean square of 0.076, with a significance level of *p* < 0.01, indicating that the model is significant. The out-of-fit term F-value is 3.22, the *p*-value is 0.1381, *p* > 0.05, indicating that the out-of-fit term is insignificant compared to the net error, indicating that the predicted and experimental values of the model are in good agreement [32]. The coefficient of determination R^2^ = 0.9931 and the corrected coefficient of determination RAdj^2^ = 0.9104 indicate that the regression model is appropriately chosen and has goodness of fit and predictive ability. Adeq Precision indicates the model’s signal-to-noise ratio, and a value greater than 4 indicates that the model is good at resisting interference [33]. The model’s Adeq Precision = 11.915, suggesting that it is accurate and reliable in the range of independent variables. In addition, the primary terms A and B and the interaction term AB in this regression model had a highly significant effect on the yields of the saikosaponins; the primary term C and the interaction term AC had a significant effect on the yields of the saikosaponins; and the secondary terms A^2^, B^2^, and C^2^ had a highly significant effect on the yields of the saikosaponins, indicating that this model can be used to predict the yields of saikosaponins.

#### 2.8.2. Response Surface Interaction Analysis

**Figure 4:**
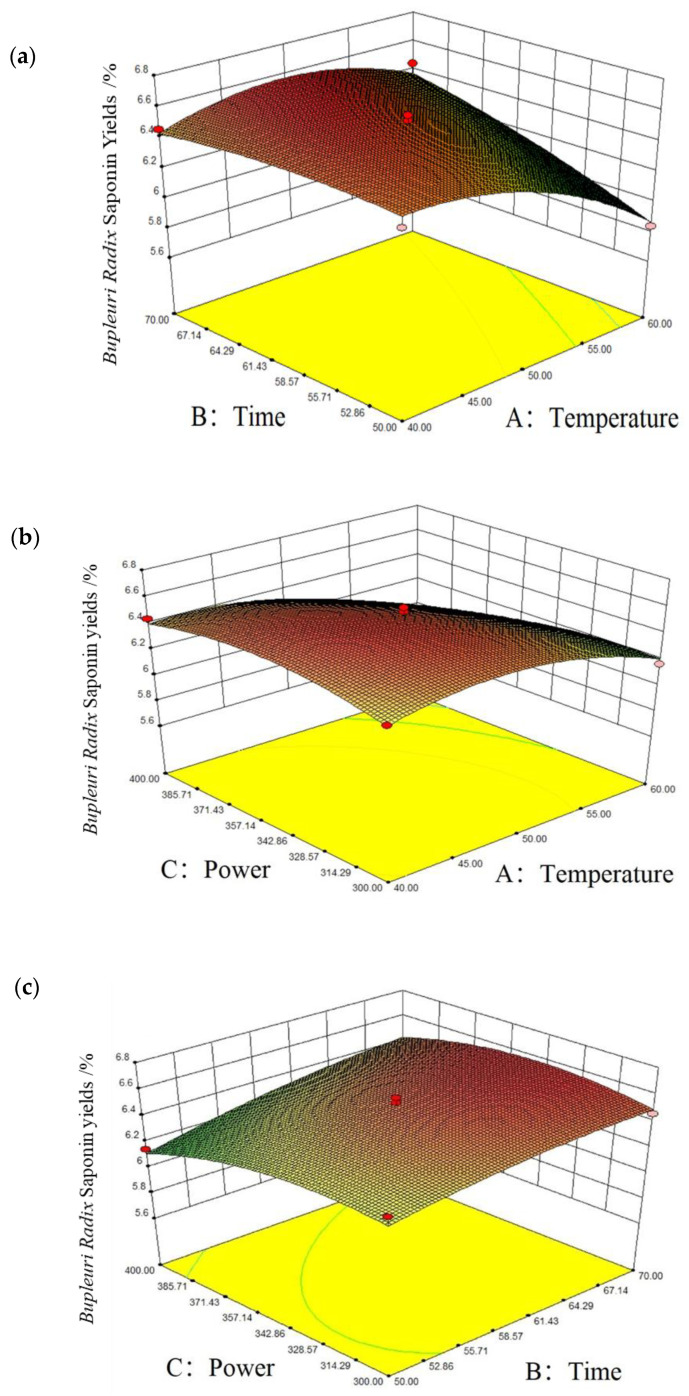
Corresponding surface plots of the interaction of various factors on the yields of saikosaponins. Notes: (**a**) is time and temperature, (**b**) is power and temperature, and (**c**) is power and time.

The significance of the interaction between the factors can be reflected by the steepness of the response surface curve: the steeper the response surface curve, the higher its effectiveness [34]. The interaction between the factors are shown in Figure 4, and Figure 4a shows the steepness of the response surface of extraction temperature > extraction time, which can indicate that the effect of the extraction temperature on the saikosaponins’ yields is more significant than the extraction time. Figure 4b shows that the power is steeper than the extraction temperature, and it can be shown that the ultrasound power > temperature. In Figure 4c, it can be seen that the steepness of the response surface of ultrasound power > extraction time, and it can be shown that the effect of ultrasound power on the saikosaponins’ yields is more significant than the extraction time. Thus, the order of influence of each factor on the yields of saikosaponins is as follows: ultrasonic power > extraction temperature > extraction time. Moreover, the order of impact of the interaction of each element on saikosaponins of *Bupleuri* Radix is as follows: BC > AB > AC.

#### 2.8.3. Prediction and Verification of Optimal Processes

The optimal process for the extraction of saikosaponins was predicted using Design-Expert.V8.0.6.1 software, and a temperature of 46.66 °C, an extraction time of 65.07 min, and an ultrasonic power of 345.56 W were obtained. The model predicted a maximum saikosaponin yield of 6.56% under these conditions. To further optimize this process and make it more suitable for practical production, we adjusted the temperature of the saikosaponin extraction process to 47 °C, the extraction time to 65 min, and the ultrasonic power to 360 W. Under these conditions, three process validation experiments were performed, and an average saikosaponin yield of 6.32% was obtained, which was in agreement with the predicted value, indicating that our optimized parameters are accurate and reliable and were able to help achieve higher yields of saikosaponins.

### 2.9. Antioxidant Activity of Saikosaponins

#### 2.9.1. Scavenging of DPPH Radicals by Saikosaponins

**Figure 5:**
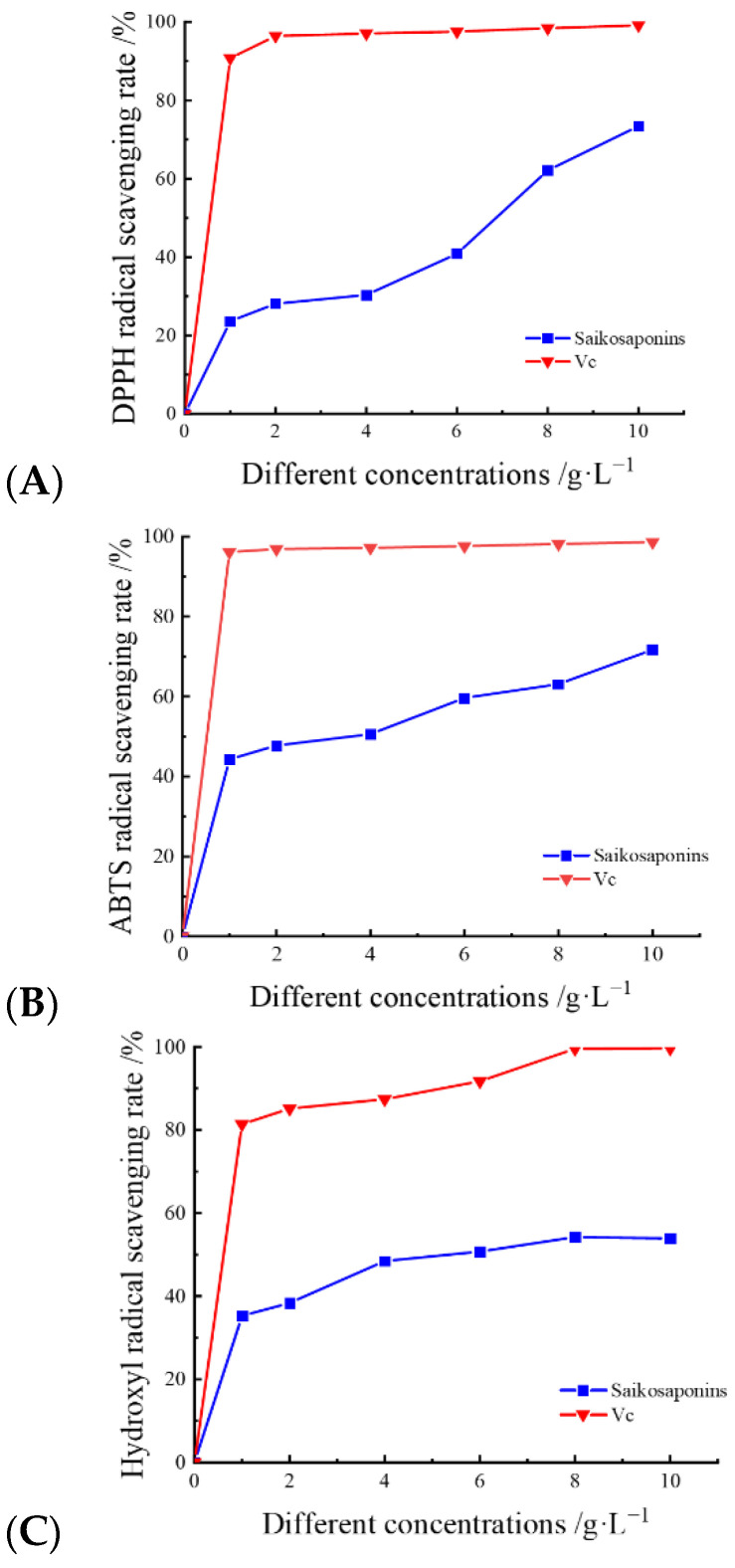
The scavenging rates of DPPH (**A**), ABTS (**B**), and -OH (**C**) radicals by saikosaponins.

The mechanism of the scavenging effect of saponins on DPPH radicals is that DPPH radicals form a stable non-radical state, DPPH-H, through hydrogen bonding provided by antioxidants, which is widely used to evaluate the scavenging effect of active ingredients on free radicals [35]. As shown in Figure 5A, the scavenging activity of saikosaponins on DPPH free radicals increased when their concentrations were increased, and the highest scavenging rate of 83.43% was achieved at a concentration of 10 g·L^−1^, which was close to that of Vc. This is similar to the results of Tan et al. [17], who studied the scavenging rate of DPPH radicals by saikosaponins under different solvent extractions. The result shows that saikosaponins have a strong scavenging ability for DPPH.

#### 2.9.2. Scavenging of ABTS Radicals by Saikosaponins

ABTS radical scavenging activity is determined using a decolorization assay for lipophilic and hydrophilic antioxidants at different pH values and has been widely used in the evaluation of antioxidant activity in vitro [36]. The ABTS radical scavenging activity is shown in Figure 5B. The radical scavenging rate of saikosaponins at concentrations of 0~8 g·L^−1^ for ABTS radicals increased gradually with the increase in concentration, and the change was relatively small. The scavenging rate of free radicals at concentrations of 8~10 g·L^−1^ reached its maximum and the change tended to be stable. The maximum scavenging rate reached 83.72%, which was close to that of Vc. The results showed that the saikosaponins extracted in this experiment had a strong scavenging ability for ABTS free radicals.

#### 2.9.3. Hydroxyl Radical Scavenging by Saikosaponins

Hydroxyl radicals are considered to be a major contributor to oxidative damage in cells and can be formed in cells through the Fenton reaction, which may lead to cellular aging and tissue damage [37]. As shown in Figure 5C, the scavenging effect of saikosaponins on hydroxyl radicals was dose-dependent. The free scavenging of -OH by saikosaponins gradually increased from 0% to 54.91% when the concentration was gradually increased from 0 g·L^−1^ to 10 g·L^−1^, and the scavenging rate was significantly lower than that of Vc. The relatively low scavenging of hydroxyl radicals by saikosaponins in comparison to DPPH and ABTS may be attributed to the fact that the hydroxyl radical scavenging assay was carried out in an acidic environment and SSa and SSd are easily affected by phenolic or acidic components, as they can be hydrolyzed and converted to SSb_1_ and SSb_2_ [38], resulting in a decrease in saikosaponins content and consequently a decrease in the scavenging rate of hydroxyl radicals. The results showed that saikosaponins have scavenging ability for hydroxyl radicals.

### 2.10. Validation of the Antioxidant Activity of Saikosaponins Using Network Pharmacology

#### 2.10.1. Prediction of Potential Targets of Saikosaponins

**Figure 6:**
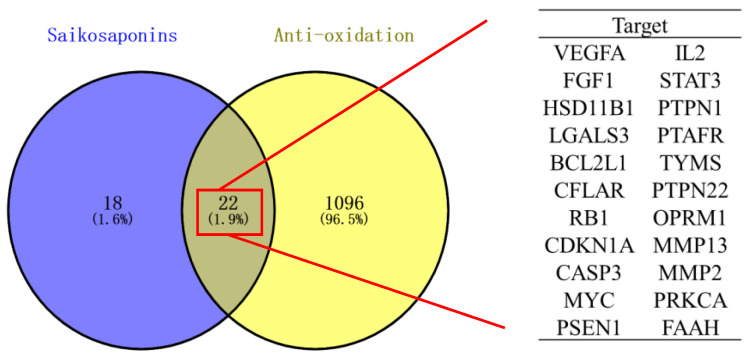
Venn diagram of saikosaponins and antioxidant effects.

Forty-one targets for the seven saikosaponins were obtained from the Swiss Target Prediction and TCMSP databases, and 1120 antioxidant-related targets were obtained by applying the OMIM and Gene Cards databases. The Venn diagram was plotted by entering the saikosaponins and antioxidant targets separately on the Venny 2.1.0 website. As shown in Figure 6, there were 22 common targets for saikosaponins and antioxidants.

#### 2.10.2. PPI Network Construction and Core Target Screening

**Figure 7:**
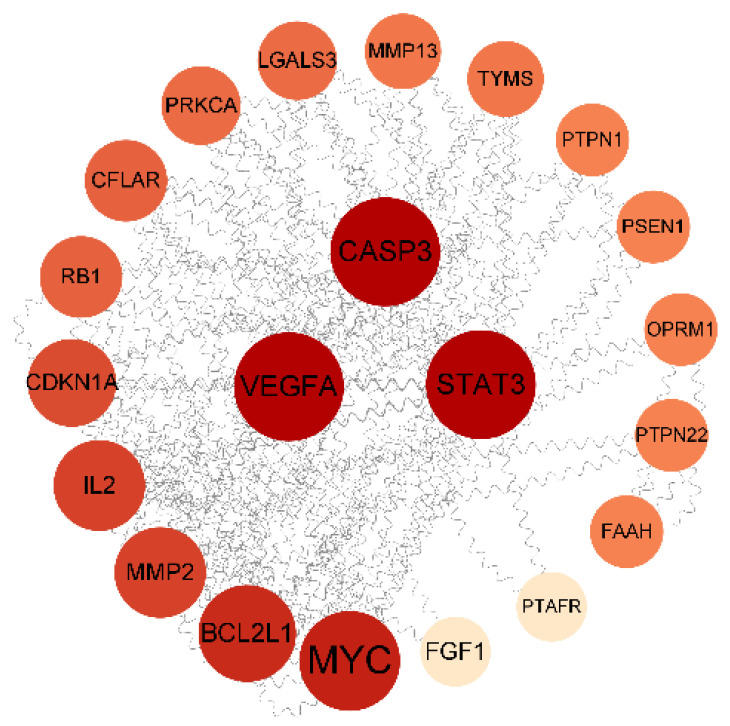
PPI network interactions diagram.

The PPI network was constructed using the STRING database. The confidence of the PPI graph of saikosaponins–antioxidants interactions was set to 0.4, which included 22 nodes and 61 edges, and the average connectivity of the nodes was 5.5. In Cytoscape_v3.9.1 software, the graph was plotted using the degree value size, where a larger degree value of the target corresponds to a larger and darker chart, which indicates that the active ingredient exerts its effect through the target. The more significant the chance of there being an antioxidant effect through the target, the greater the chance of there being an antioxidant effect. As shown in Figure 7, saikosaponins interact with 28 proteins with the highest degree of CASP3, VEGFA, and STAT3 among the core targets of antioxidant action, where the expression of the target VEGFA affects IPEC-J2 cell proliferation, cell cycle progression, and ROS content and alters the levels of antioxidant enzymes and inflammatory factors [39]. CASP3 is involved in the apoptosis process, where hypoxia and nutrient deficiency can significantly increase apoptosis [40]. STAT3 is involved in the expression of various genes, such as cell proliferation, survival, differentiation, migration, angiogenesis, and inflammation [41]. The average value of BC, CC, and DC was used as the screening condition for the core target screening. Table 5 shows that the core targets of saikosaponins and antioxidants are CASP3, VEGFA, STAT3, MYC, BCL2L1, and IL2.

#### 2.10.3. GO Gene Function and KEGG Pathway Analysis

**Figure 8:**
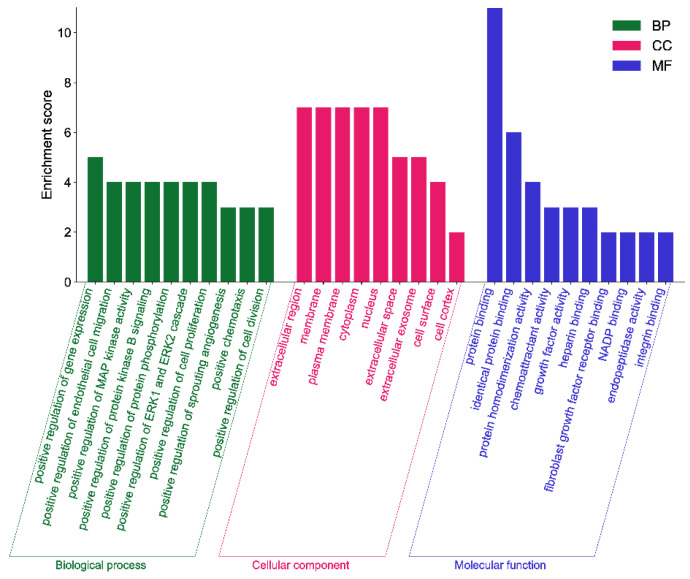
GO enrichment analysis of saikosaponins–antioxidants intersection targets.

**Figure 9:**
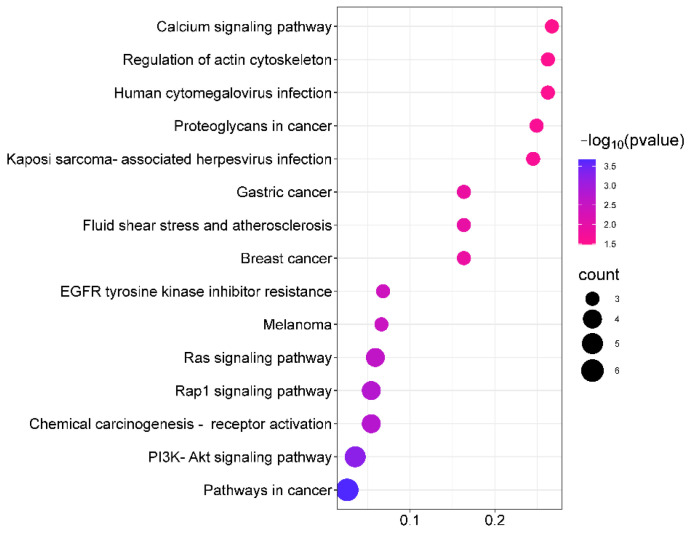
Analysis of KEGG pathway between saikosaponins and antioxidant- related targets.

The GO analysis results show that the saikosaponins and antioxidant target GO enrichment obtained a total of 155 targets, including 115 BPs, 19 CCs, and 21 MFs, all of which were selected for GO enrichment analysis bar graphs with *p* < 0.05 and a count number ranked in the top 10, see Figure 8. Saikosaponins exert antioxidant effects, involving a variety of BPs, CCs, and MFs. For example, the biological processes involve the negative regulation of apoptosis, negative regulation of gene expression, the positive regulation of gene expression, protein binding, endopeptidase activity, and cysteine-type endopeptidase activity involved in the apoptosis process. As shown in Figure 9, 51 pathways are obtained by saikosaponins enrichment (*p* < 0.05), and the count value of the top 15 KEGG signaling pathways are selected to create bubble plots using KEGG enrichment analysis. The pathways enriched by saikosaponins are mainly the cancer pathway, the PI3K-Akt signaling pathway, the Hepatitis C/B pathway, the JAK-STAT signaling pathway, Epstein–Barr virus infection, etc. Among them, the PI3K/Akt signaling pathway can inhibit Nrf2/HO-1 activation and effectively protect cells from UVB radiation-induced oxidative stress [42]. JAK-STAT can be involved in inhibiting cancer tumor effects [43], and studies have shown that antioxidants are frequently used in cancer therapy [44]. Thus, it can be seen that the BPs, CCs, MFs, and KEGG pathways enriched by the interaction of saikosaponins with antioxidants, which are closely related to the antioxidant stress response, indicate that saikosaponins can counteract the antioxidant stress response of the body through various pathways.

#### 2.10.4. Molecular Docking Verification

**Figure 10:**
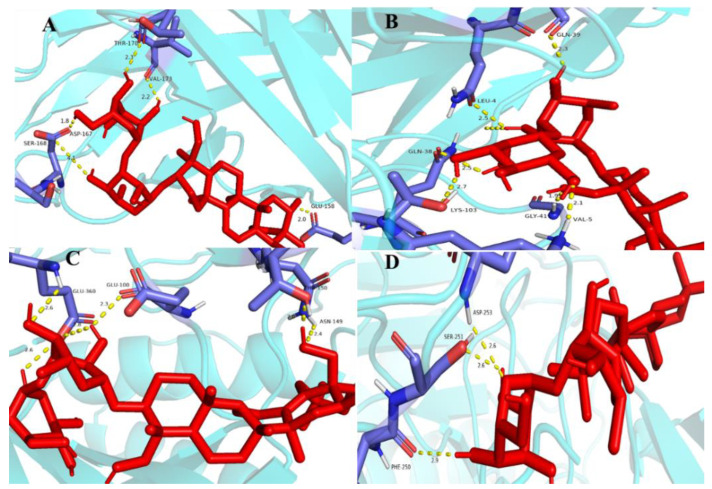
Molecular docking results: graph (**A**) is SSa and VEGFA, (**B**) is SSb_2_ and VEGFA, (**C**) is SSb_1_ and STAT3, (**D**) is SSe and CASP3. Notes: The red colour in the figure represents the structure of antioxidant proteins and the blue colour represents the structure of saponin targets.

Molecular docking is a technique for predicting the connectivity of ligand compounds to known three-dimensional structured proteins. In the present study, seven saikosaponins (SSa, SSb_1_, SSb_2_, SSc, SSd, SSe, and SSf) were docked to the core targets CASP3, STAT3, and VEGFA. It was shown that the lower the molecular docking binding energy, the stronger the binding ability between the molecule and the target, indicating that these compounds bind strongly to the protein [45]. Binding energies less than −5.0 kcal·mol^−1^ usually indicate a good binding activity between donor and acceptor, while binding powers less than −7.0 kcal·mol^−1^ indicate stronger binding activity [46]. As shown in Table 6, the seven saikosaponins docked with CASP3, STAT3, and VEGFA, except for SSf with a STAT3 docking binding energy of −6.3 kcal·mol^−1^, had docking scores less than −7.0 kcal·mol^−1^, indicating that the components of saikosaponins have the strong binding ability with the target proteins of antioxidant action. The selected docking binding energy was smaller for SSa with VEGFA, SSb_2_ with VEGFA, SSb_1_ with STAT3, and SSe with CASP3, and these bindings were chosen to make the graphs. It can be seen from Figure 10 that the compounds have better connectivity and stronger binding ability with the targets, which further determines the antioxidant properties of saikosaponins.

## 3. Materials and Methods

### 3.1. Plant Material

*Bupleuri* Radix tablets were purchased from Gansu Longxi Qizheng medicinal materials Co., Ltd. (Longxi, China). The content of the tablets was identified as the dried roots of the plant *Bupleuri Chinese* DC. by Professor Yuan Chen of the College of Agronomy, Gansu Agricultural University.

### 3.2. Chemicals

Methanol (chromatographic grade), acetonitrile (chromatographic grade), ferrous sulfate, potassium persulfate ≥ 99.5, trichloroacetic acid ≥ 99%, hydrogen peroxide 30%, L(+)-ascorbic acid (VC), and salicylic acid were purchased from Sinopharm Group Chemical Reagent Co., Ltd. (Shanghai, China); SSb_1_ ≥ 99.37%, SSb_2_ ≥ 98.99%, SSc ≥ 99.90%, SSd ≥ 98.56%, SSe ≥ 94.545%, and SSf ≥ 98.89% were purchased from Chengdu Pfeiffer Biotechnology Co., Ltd., (Shanghai, China); and 2,2′-hydrazine-bis(3-ethylbenzothiazoline-6-sulfonic acid) diamine salt (ABTS) ≥ 98% was purchased from Beijing Solabao Technology Co., (Beijing, China).

### 3.3. Pre-treatment of Bupleuri Radix Herb

The *Bupleuri* Radix tablets were baked at 40 °C for 2 h and then crushed in a small high-speed crusher, and the herbs were sieved through a No. 4 sieve to produce the crude powder of *Bupleuri* Radix, which was packed into a self-sealing bag and stored in a refrigerator at 4 °C as a backup.

### 3.4. Preparation of Sample Solution

The sample solution was prepared by referring to the method of Mo et al. [47], with slight modification. First, 1 g of the powder was weighed and placed in a conical flask with a stopper. Then, 20 mL of solvent was added and mixed well and weighed with the plug closed. The extract was placed in an ultrasonic pot with an ultrasonic power of 200 W and temperature of 30 °C for 30 min, then cooled to room temperature, weighed, and the lost weight was made up using the extraction solvent, shaken well, and centrifuged for 15 min. The supernatant was removed, passed through a 0.45 μm microporous filter membrane, stored at 4 °C, and set aside. Each test was averaged three times, and the average value was taken.

### 3.5. Establishment of HPLC-DAD Dual Wavelength Method for the Determination of Saikosaponins

#### 3.5.1. Preparation of Standard Solutions

**Figure 11:**
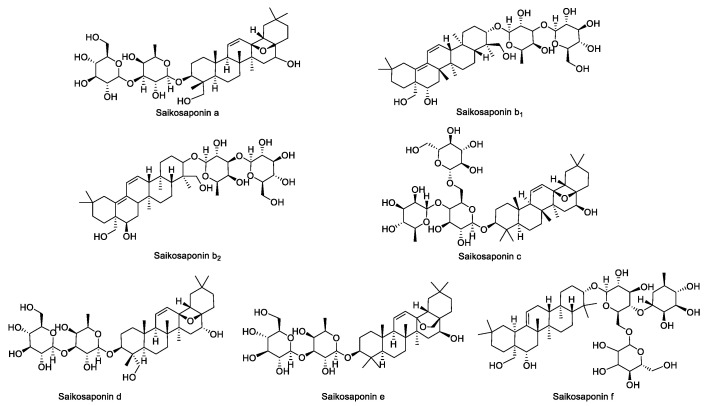
The structural formulae of the seven saikosaponins.

The standards of SSa, SSb_1_, SSb_2_, SSc, SSd, SSe, and SSf were configured into 1 mg·mL^−1^ solutions. Then, each standard was precisely aspirated in appropriate amounts to be configured into concentrations of 0.05 mg·mL^−1^, 0.1 mg·mL^−1^, 0.2 mg·mL^−1^, 0.3 mg·mL^−1^, 0.4 mg·mL^−1^, 0.45 mg·mL^−1^, 0.5 mg·mL^−1^ of the mixed standard solution, and the molecular formulae of the seven saikosaponins are shown in Figure 11.

#### 3.5.2. Determination of the Detection Method

The HPLC-DAD dual wavelength method was used to simultaneously determine the content of the seven saikosaponins, and the chromatographic conditions referred to the method [38] of Liu et al. A Symmetry-C_18_ column (4.6 mm × 250 mm, 5 μm) was used with a column temperature of 30 °C, the mobile phase was acetonitrile–water, the flow rate was 1.0 mL·min^−1^, the injection volume was 8 μL, and the detection wavelengths were 210 nm and 254 nm. The mobility elution method is shown in Table 7.

#### 3.5.3. Methodological Validation

The method’s linearity, precision, stability, and accuracy were verified [12] under HPLC-DAD conditions. The linearity was checked by three analyses of seven mixed standard solutions at different concentrations, and the calibration curves were constructed via linear regression analysis of integrated peak area (y) versus concentration (x), and the intraday precision was evaluated by injecting the mixed standard solution 6 times in one day. The stability of the method was evaluated continuously by measuring the sample solution 6 times. The strength of the process solution and the instrument was assessed by determining the mixed standard solution at different times (0 h, 3 h, 6 h, 10 h, 15 h, 24 h).

### 3.6. Determination of Optimal Extraction Conditions for Saikosaponins

#### 3.6.1. Determination of the Most Suitable Extraction Solvent

Methanol, water, anhydrous ethanol, and 5% ammonia–methanol solution were used as solvents to extract saikosaponins, and the best extraction solvent was determined by the size of the sum of the seven saikosaponins’ yields.

#### 3.6.2. Selection of Optimal Ultrasonic Extraction Conditions

The extracts were analyzed under the optimized chromatographic conditions, and the sum of the seven saikosaponins were used to select the extracts. The optimum temperature (20~70 °C), ultrasonic power, extraction time, and material-to-liquid ratio were selected based on the sum of the seven saikosaponins. The average value was taken by repeating each test three times.

### 3.7. Response Surface Optimization of the Extraction Process

Based on the single-factor experiment with extraction time, ultrasonic power, and temperature as independent variables and the yields of saikosaponins as the response value of the investigation, a three-factor, three-level response surface optimization experiment was conducted, as shown in Table 8. The average values were taken by measuring each experiment three times.

### 3.8. Determination of Antioxidant Activity of Saikosaponins

#### 3.8.1. Determination of DPPH Free Radical Scavenging Activity of Saikosaponins

**Figure 12:**
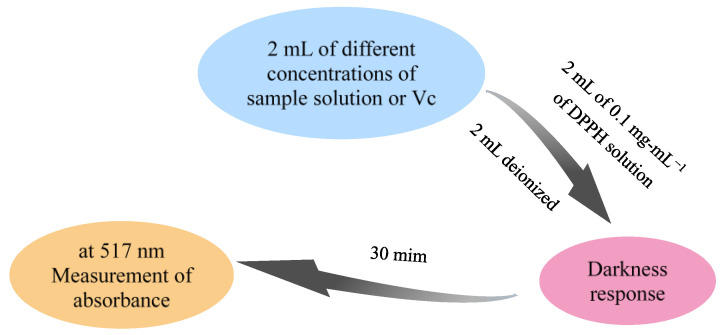
Flow chart of DPPH free radical scavenging test.

The method for determining scavenging DPPH radical activity was based on the method [48] of Sun et al., with slight modifications, and the experimental flow chart is shown in Figure 12. The DPPH radical cation scavenging activity was calculated according to Equation (1) using Vc as a positive control:

(1) $$\mathrm{DPPH}\;\mathrm{free}\;\mathrm{radical}\;\mathrm{scavenging}\;\mathrm{rate}=\left[1-\frac{A_{1}-A_{2}}{A_{0}}\right]\times 100\%$$

where A_0_ is the absorbance of the blank control DPPH solution, A_1_ is the absorbance of the sample in DPPH solution, and A_2_ is the absorbance of the sample solution.

#### 3.8.2. Determination of Hydroxyl Radical Scavenging Activity of Saikosaponins

**Figure 13:**
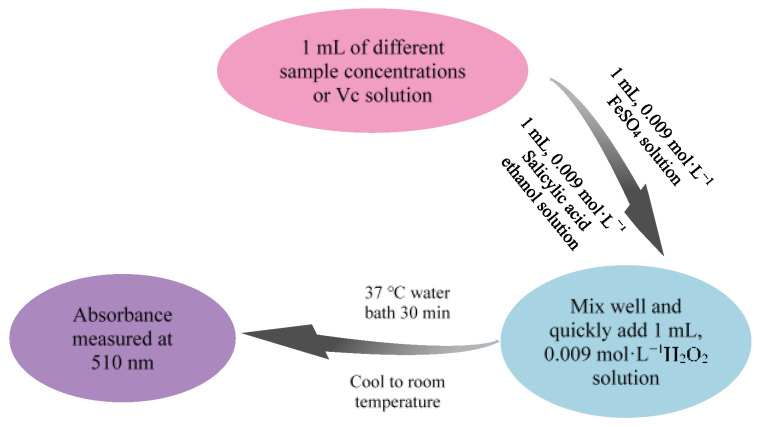
Flow chart of hydroxyl radical scavenging experiment.

The experimental method for scavenging hydroxyl radicals is referenced from Chen et al. [49], with slight modifications. The experimental flow chart is shown in Figure 13. Vc was used as a positive control. The clearance rate was calculated as described in Equation (2).

(2) $$\mathrm{Hydroxyl}\;\mathrm{radicals}\;\mathrm{free}\;\mathrm{radical}\;\mathrm{scavenging}\;\mathrm{rate}=\left[1-\frac{C_{1}-C_{2}}{C_{0}}\right]\times 100\%$$

in the formula, C_0_ is no sample solution absorption, C_1_ is added to the sample solution’s absorption, and C_2_ is the absorption of the sample solution itself.

#### 3.8.3. Determination of the Free Radical Scavenging Activity of Saikosaponins against ABTS

**Figure 14:**
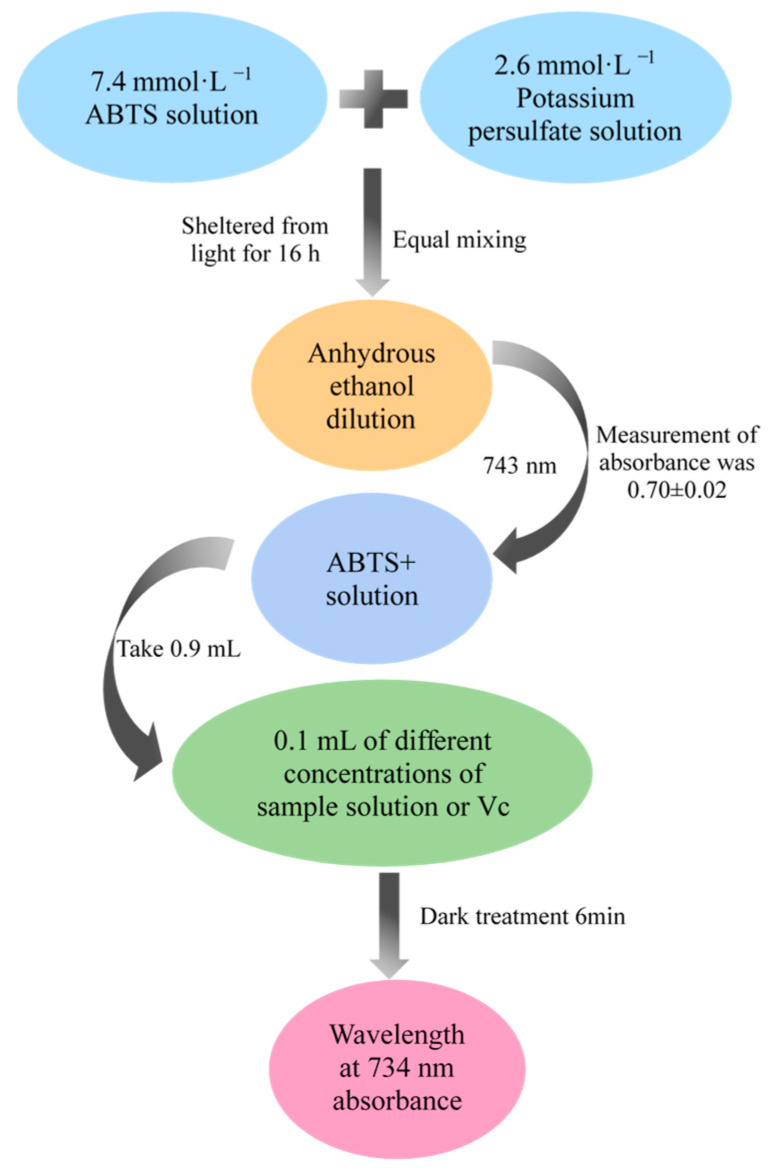
Flow chart of ABTS free radical scavenging experiment.

The method for scavenging ABTS radicals was referenced from Wang et al. [50], with slight modifications. The experimental flow chart is shown in Figure 14. The cation scavenging activity of ABTS radicals was calculated according to Equation (3) using Vc as a positive control:

(3) $$\mathrm{ABTS}\;\mathrm{free}\;\mathrm{radical}\;\mathrm{scavenging}\;\mathrm{rate}=\left[1-\frac{B_{1}}{B_{0}}\right]\times 100\%$$

in the formula, B_1_ is the mixture of ABTS and the sample and B_0_ is the mixture of ABTS and water.

### 3.9. The Antioxidant Mechanism of Saikosaponins Based on Network Pharmacology

#### 3.9.1. Prediction of the Targets Corresponding to the Active Components of the Seven Saikosaponins

The saikosaponins extracted and analyzed in this work were imported into the TCMSP (https://old.tcmsp-e.com/tcmsp.php, accessed on 18 April 2023) database [51], and the PubChem database (https://pubchem.ncbi.nlm.nih.gov, accessed on 18 April 2023) and Swiss Target Prediction (http://www.swisstargetprediction.ch/, accessed on 18 April 2023) databases [52,53] were used to collect the targets of saikosaponins. The obtained target information was imported into the UniProt (https://www.uniprot.org, accessed on 18 April 2023) database for transformation [54] to obtain the targets of the seven saikosaponins.

#### 3.9.2. Disease Target Acquisition

The required targets for antioxidants were collected in OMIM data (https://www.omim.org, accessed on 25 April 2023) [55,56] and GeneCards database (https://www.genecards.org, accessed on 25 April 2023) [57].

#### 3.9.3. PPI Network Construction and Core Target Acquisition

On the Venny 2.1.0 (www.liuxiaoyuyuan.cn, accessed on 25 April 2023) website, the intersection targets of the interaction between saikosaponins and antioxidants were obtained and then entered into the STRING database (https://string-db.org, accessed on 25 April 2023) [58] to obtain a PPI network diagram. Finally, the intermediary centrality, betweenness centrality (BC), closeness centrality(CC), degree centrality (DC), and screen core targets were calculated in Cytoscape.

#### 3.9.4. GO Function and Module Analysis, KEGG Enrichment Pathway Analysis

The intersecting targets of saikosaponins and antioxidant effects were entered into the DAVID 2021 (https://david.ncifcrf.gov, accessed on 28 April 2023) [59,60] website for GO and KEGG pathway analysis.

#### 3.9.5. Molecular Docking

Seven saikosaponins were molecularly docked to the core targets. First, the compounds were downloaded from the TCSMP database in mol2 format, and the protein PDB files were retrieved from the PDB database. The protein PDB files were imported into Pymol, and the “remove solvent” and “remove organic” functions were entered to remove water and small molecules. Next, hydrogenation and charge calculations were performed on the protein using AutoDock Vina 1.5.7. in the results were saved in pdbqt format and the compound converted from mol2 format to pdbqt format. Then, the docking position for molecular docking was set, and the graphical analysis was performed in pymol 2.5.0.

### 3.10. Data Processing

Excel 2016, Design Expert 8.0.6.1, and Origin 2021 were used to process the experimental data and perform statistical analysis.

## 4. Conclusions

In this study, we found that SSa, SSc, SSd, SSe, and SSf were better separated at the wavelength of 210 nm, and SSb_1_ and SSb_2_ were better separated at 254 nm under the dual wavelength of HPCL-DAD, which indicates that the chromatographic conditions could be separated well for the seven major saikosaponins. The yields of the seven saikosaponins were 6.32% under the optimal conditions optimized by Box–Behnken experimental design. This study further validated the antioxidant mechanism of saikosaponins. The seven saikosaponins can interact with antioxidant targets and participate in various antioxidant biological pathways, and their binding energies to the CASP3, STAT3, and VEGFA target molecules are less than −5.0 kcal·mol^−1^. This study provides a new method for efficiently utilizing the saikosaponins in *Bupleuri* Radix and provides a theoretical basis for using saikosaponins as natural antioxidants.

## Figures and Tables

**Table 1:** Linearity equation and linear range.

Ingredient	Regression Equation	Linear Range /μg·mL^−1^	R^2^
SSa	y = 2.3811x − 2.5163	7.413~71.413	R^2^ = 0.9991
SSc	y = 0.7631x − 0.0868	7.413~71.413	R^2^ = 0.9991
SSd	y = 1.1456x − 2.0674	7.413~71.413	R^2^ = 0.9997
SSe	y = 1.0351x − 1.9814	7.413~71.413	R^2^ = 0.9996
SSf	y = 0.8913x − 1.0791	7.413~71.413	R^2^ = 0.9992
SSb_1_	y = 9.9591x − 11.583	7.413~71.413	R^2^ = 0.9994
SSb_2_	y = 7.1208x − 4.8388	7.413~71.413	R^2^ = 0.9992

**Table 2:** RSD values of precision, stability, and reproducibility of seven saikosaponins.

Compound	Precision Density/%	Repeatability/%	Stability/%
SSa	2.67	1.03	0.41
SSc	1.34	0.10	0.29
SSd	0.55	0.23	1.27
SSe	1.75	1.79	2.43
SSf	0.66	0.53	1.43
SSb_1_	1.39	0.22	0.66
SSb_2_	0.89	0.15	1.52

**Table 3:** Box–Behnken experimental design and the yields of saikosaponins.

No.	Factors			Saikosaponin Yields/%
	A—Temperature (°C)	B—Time (min)	C—Power (W)	
1	60	50	350	5.843
2	50	70	400	6.366
3	60	70	350	6.444
4	40	70	350	6.458
5	50	50	400	6.153
6	60	60	300	6.172
7	50	70	300	6.458
8	50	60	350	6.559
9	60	60	400	5.885
10	50	50	300	6.407
11	50	60	350	6.468
12	50	60	350	6.525
13	50	60	350	6.525
14	40	50	350	6.412
15	40	60	300	6.375
16	40	60	400	6.440
17	50	60	350	6.437

**Table 4:** Analysis of variance table for the quadratic model.

Source of Variance	Sum of Squares	Degree of Freedom	Mean Square Sum	F-Value	*p*-Value	Significance
Models	0.68	9	0.076	15.81	0.0007	**
A—Temperature	0.22	1	0.22	46.66	0.0002	**
B—Time	0.1	1	0.1	21.55	0.0024	**
C—Power	0.04	1	0.04	8.38	0.0232	*
AB	0.07	1	0.077	16.06	0.0051	**
AC	0.03	1	0.031	6.39	0.0393	*
BC	6.58 × 10^−3^	1	6.58 × 10^−3^	1.37	0.2805	
A^2^	0.12	1	0.12	25.57	0.0015	**
B^2^	7.64 × 10^−3^	1	7.64 × 10^−3^	1.59	0.248	
C^2^	0.055	1	0.055	11.37	0.0119	*
Residuals	0.034	7	4.81 × 10^−3^			
Loss of proposed items	0.024	3	8.02 × 10^−3^	3.32	0.1381	not significant
Pure error	9.64 × 10^−3^	4	2.41 × 10^−3^			
Total deviation	0.72	16				

Note: “*” indicates significant difference (*p* < 0.05); “**” indicates extremely significant difference (*p* < 0.01); *p* > 0.05 indicates not significant.

**Table 5:** Information and topological properties of PPI network diagrams for the interaction of saikosaponins with antioxidants.

Compounds	Targets	BC	CC	DC
Saikosaponins	CASP3	58.60	0.74	28
	VEGFA	83.23	0.74	28
	STAT3	80.26	0.74	28
	MYC	20.43	0.66	22
	BCL2L1	21.43	0.64	20
	IL2	103.0	0.62	16

**Table 6:** Binding energy of key targets docked with active ingredients.

Compound	Combined Energy/kcal·mol^−1^		
	STAT3	VEGFA	CASP3
SSa	−7.4	−9.2	−8.1
SSb_1_	−8.0	−8.6	−8.0
SSb_2_	−7.6	−9.5	−7.4
SSc	−7.8	−9.1	−7.6
SSd	−7.9	−9.2	−7.9
SSe	−7.7	−9.1	−8.7
SSf	−6.3	−8.0	−7.7

**Table 7:** Gradient elution schedule for chromatographic conditions.

Time/min	Mobile Phase Ratio/%	
	Acetonitrile	Water
0	33	67
20	39	61
40	53	47
50	33	67

**Table 8:** Response surface test factors and levels.

Variables	Level		
	−1	0	1
A—Temperature/°C	40	50	60
B—Time/min	50	60	70
C—Ultrasonic power/W	300	350	400

## Data Availability

The data presented in this study are available on request from the corresponding author.

## Sample Availability

Samples of the plant materials of *Bupleuri Chinese DC.* are available from the authors.
